# Structure determination of an amorphous drug through large-scale NMR predictions

**DOI:** 10.1038/s41467-021-23208-7

**Published:** 2021-05-20

**Authors:** Manuel Cordova, Martins Balodis, Albert Hofstetter, Federico Paruzzo, Sten O. Nilsson Lill, Emma S. E. Eriksson, Pierrick Berruyer, Bruno Simões de Almeida, Michael J. Quayle, Stefan T. Norberg, Anna Svensk Ankarberg, Staffan Schantz, Lyndon Emsley

**Affiliations:** 1 Institut des Sciences et Ingénierie Chimiques, École Polytechnique Fédérale de Lausanne (EPFL), Lausanne, Switzerland; 2 National Centre for Computational Design and Discovery of Novel Materials MARVEL, École Polytechnique Fédérale de Lausanne (EPFL), Lausanne, Switzerland; 3 Early Product Development and Manufacturing, Pharmaceutical Sciences, R&D, AstraZeneca, Gothenburg, Sweden; 4 New Modalities and Parenteral Development, Pharmaceutical Technology & Development, Operations, AstraZeneca, Gothenburg, Sweden; 5 Oral Product Development, Pharmaceutical Technology & Development, Operations, AstraZeneca, Gothenburg, Sweden

**Keywords:** Solid-state NMR, Molecular dynamics, Structure prediction

## Abstract

Knowledge of the structure of amorphous solids can direct, for example, the optimization of pharmaceutical formulations, but atomic-level structure determination in amorphous molecular solids has so far not been possible. Solid-state nuclear magnetic resonance (NMR) is among the most popular methods to characterize amorphous materials, and molecular dynamics (MD) simulations can help describe the structure of disordered materials. However, directly relating MD to NMR experiments in molecular solids has been out of reach until now because of the large size of these simulations. Here, using a machine learning model of chemical shifts, we determine the atomic-level structure of the hydrated amorphous drug AZD5718 by combining dynamic nuclear polarization-enhanced solid-state NMR experiments with predicted chemical shifts for MD simulations of large systems. From these amorphous structures we then identify H-bonding motifs and relate them to local intermolecular complex formation energies.

## Abstract (web summary)

Determining the structure of amorphous solids is important for optimization of pharmaceutical formulations, but direct relation of molecular dynamics (MD) simulations and NMR to achieve this is challenging. Here, the authors use a machine learning model of chemical shifts to solve the atomic-level structure of the hydrated amorphous drug AZD5718 by combining dynamic nuclear polarization-enhanced solid-state NMR with predicted shifts for MD simulations of large systems.

## Introduction

Determination of the structure of organic solids is a key step in many areas of chemistry, and in particular for the design of efficient and safe pharmaceutical compounds. This requires methods for atomic-level structure determination. Whereas X-Ray diffraction (XRD) is the established gold standard when single crystals are available, atomic-level structure determination is much more challenging in powder samples, and even more so if the structures are disordered. Indeed, while there has been much progress towards complete structure determination in crystalline powders, by powder XRD^1,2^ or particularly from solid-state nuclear magnetic resonance (NMR) approaches^3–5^, the disorder inherent to amorphous solids makes structure determination elusive.

For example, the structure, accessible surface area, and stability of amorphous drug formulations are of high current interest^6–11^, particularly since the bioavailability or dissolution rate of poorly soluble compounds in crystalline forms is often a severe limitation on the chemical space available for development of active pharmaceutical ingredients (APIs), and since the uptake of poorly soluble drugs can be significantly enhanced in amorphous formulations. In particular hydrated amorphous phases, wherein water molecules closely interact with the drug through hydrogen bonds, have been investigated for several systems of pharmaceutical interest^12–14^.

Solid-state NMR is among the most popular methods to study the structure of amorphous materials. While two-dimensional correlation experiments are able to identify intermolecular contacts between atom pairs^15–17^, obtaining complete atomic-level structures is a challenge due to the disordered molecular environments present in amorphous solids.

Comparing chemical shifts obtained from solid-state NMR experiments with shifts computed using density functional theory (DFT) methods for trial structures generated using crystal structure prediction (CSP) or molecular dynamics (MD) approaches has proven able to determine the structures of powdered crystalline organic (and hybrid) solids^3,18,19^. One limitation of this procedure lies in the accuracy achievable by the DFT method used. While plane-wave gauge-including projector augmented wave (GIPAW) is the most popular approach to computing chemical shifts in crystalline solids^20,21^, the increased computational cost incurred by the use of a plane-wave basis set instead of an atomic orbital basis set typically limits the level of theory to generalized gradient approximation (GGA) functionals. An alternative fragment- and cluster-based approach involving atomic orbital basis sets has recently been developed by Hartman et al. and allows the use of hybrid functionals, increasing the accuracy of computed chemical shifts at a computational cost comparable to GIPAW^22–25^.

Candidate structures of amorphous compounds can be generated using MD simulations^26–28^, however the large cell sizes required to model amorphous structures, together with the large number of structures needed to describe amorphous ensembles, have so far prevented the computation of chemical shifts in these systems. Recently a new approach to predicting chemical shifts in molecular solids was introduced^29^ by training a machine-learning model on GIPAW DFT calculated chemical shifts for 3546 diverse structures from the Cambridge structural database (CSD)^30^. This model, dubbed ShiftML, can now be used to predict chemical shifts for any molecular solid (currently limited to solids comprised of C, H, N, O, S atoms) in a matter of seconds^29,31^, which opens up the possibility for large-scale shift computations on large structures.

Here, we investigate the structure of anhydrous crystalline AZD5718 (**1**) form A, by combining measured ^1^H, ^13^C and ^15^N chemical shifts obtained using DNP-enhanced NMR experiments from a powder sample, CSP, and DFT chemical shift computations. The structure is validated with that obtained from single-crystal XRD. We then model the hydrated amorphous drug with different water contents using MD simulations and obtain predicted NMR spectra for large structural ensembles using machine learned chemical shifts. We then analyse the ensembles to identify the different hydrogen bonding motifs present in the amorphous structures, by comparison of the experimental and predicted chemical shift distributions associated with these structural motifs. From the amorphous structures we also compute the interaction energy between molecules of **1** and their environment, and we relate the energies to the local hydrogen bonding motifs.

## Results

### NMR crystallography

**Fig. 1:**
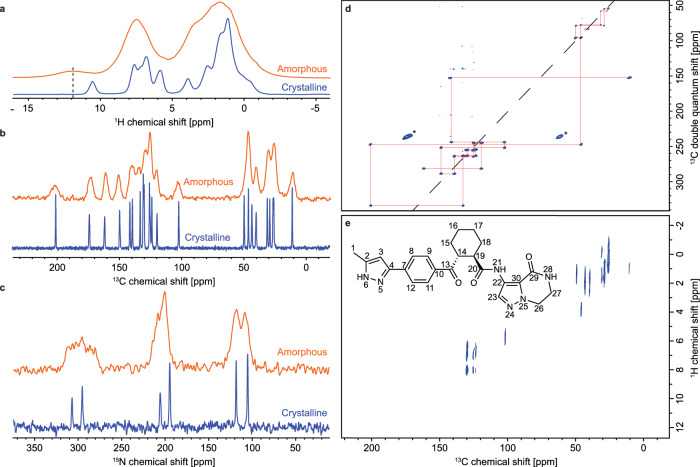
Solid-state NMR experiments. **a** ^1^H, **b** ^13^C and **c** ^15^N MAS NMR spectra of crystalline (blue) and amorphous (orange) **1**. **d** ^13^C-^13^C DNP enhanced solvent suppressed INADEQUATE and **e**, ^1^H-^13^C HETCOR spectra of crystalline **1**. The dashed black line in (**a**) indicates the chemical shift assigned to the proton bound to N6 in the amorphous sample. In (**d**), the ^13^C peaks denoted by a star at 60 and 170 ppm are attributed to impurities introduced during the NMR sample preparation. The chemical structure and labelling scheme of **1** is shown in (**e**).

**Fig. 2:**
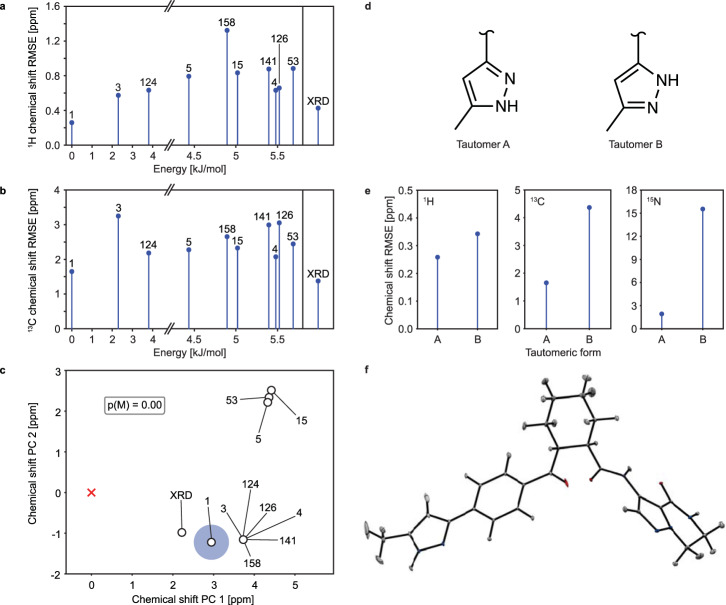
NMR crystallography of AZD5718. **a** ^1^H and **b** ^13^C chemical shift RMSEs of the ten lowest DFT-D energy CSP candidates and of the crystal structure determined by X-ray diffraction (labelled XRD). The label rank in terms of increasing force-field energy per molecule of (**1**) of each candidate is indicated above each point. **c** Two-dimensional projection of the similarity of the computed ^1^H and ^13^C chemical shifts of the candidate structures to the experimental data (red cross). The probability of each candidate to match experiment is represented by the area of the blue disk. *p(M)* denotes the probability that a virtual candidate, which represents structures potentially missing from the CSP candidate pool, matches experiment. The virtual candidate is represented by the mean of the shifts of all CSP candidates, and a high probability *p(M)* suggests that the CSP candidate pool may not include the experimentally observed structure. **d** Chemical structures of the two tautomers of **1** considered, labelled as A and B. **e** Agreement between ^1^H, ^13^C and ^15^N experimental and DFT computed chemical shifts for the two tautomers. **f** ORTEP plot of the ADP tensors for the NMR structure of **1** drawn at the 90% probability level.

No polymorphism was observed for anhydrous crystalline **1**, nor were crystalline hydrates identified. The crystal structure of anhydrous crystalline **1** Form A was determined using a chemical shift-based NMR crystallographic approach. This involves the combination of the assigned experimental chemical shifts with CSP and computed chemical shifts. The ^1^H, ^13^C and ^15^N resonances of AZD5718 (Fig. 1e) were assigned using one-dimensional proton, carbon and nitrogen MAS NMR experiments (Fig. 1a–c), as well as two-dimensional refocused ^13^C-^13^C INADEQUATE and ^1^H-^13^C HETCOR experiments (Fig. 1d-e) as detailed in Supplementary Discussion. A set of DFT-D optimized candidate structures was generated using an internally developed rapid CSP approach, then the assigned experimental chemical shifts were compared to the shifts computed using the cluster- and fragment-based DFT approach introduced by Hartman et al.^22–24^ for each structure in order to determine the experimental structure from the set of candidates. The structure determined using single-crystal X-ray diffraction was included and compared to the CSP set. The lowest energy CSP candidate (structure #1) was found to be structurally similar to the X-ray structure (as discussed in Supplementary Discussion). The comparisons of the experimental and computed ^1^H and ^13^C chemical shifts are shown in Fig. 2a, b, respectively. The root-mean-square errors (RMSEs) obtained for ^1^H suggested that structure #1 best matches the experiment, while ^13^C chemical shift results identified the X-ray structure as the best match. In addition, the DFT-D energy per molecule of structure #1 was found to be the lowest among the CSP set (x-axis in Fig. 2a-b). This also indicates that the force field used for the CSP procedure accurately describes the crystalline system, and supports the identification of candidate #1 as being the crystal structure. In order to elucidate the ambiguity between candidate #1 and the XRD structure, and to obtain a quantitative comparison of all candidates, a Bayesian probabilistic analysis was carried out using the approach introduced by Engel et al.^31^. The two main advantages of using this method to determine the structure that best matches experiment are the quantitative determination of the confidence in the identification of the experimental structure on a continuous scale from 0 to 100%, and the combined use of NMR results for several elements, increasing the accuracy of the identification.

Figure 2c shows the results obtained with the Bayesian approach, represented as a principal component analysis (PCA) plot. This plot is a two-dimensional representation of the similarity of the different candidate structures according to their computed chemical shifts. The computed Bayesian probability of each structure to be the experimental crystal structure is represented by the area of the blue disk around each point. Using both ^1^H and ^13^C chemical shifts, candidate #1 is found to be the most probable crystal structure, with 99.7% confidence. Although the structure determined by X-ray diffraction (labelled XRD) appears closer to the experimental results (red cross) in the first two chemical shift principal components in Fig. 2c, including the complete chemical shift space identifies candidate #1 as the structure that best matches experiment, as indicated by its associated confidence. Figure 2c highlights the similarity of the selected structures in terms of their chemical shifts, in the two dimensions that display the largest variance.

Comparison of the structures determined via XRD and NMR crystallography yielded a RMSD_15_ (root mean square deviation of the atomic positions in 15 molecules, ignoring hydrogen positions) of 0.42 Å. The main difference between the two structures lies in the conformation of the bicyclo ring (see Supplementary Fig. 2). Single-molecule heavy atom RMSD was found to be 0.22 Å, and was decreased to 0.15 Å after omitting the two carbons of the bicyclo ring (labelled 26 and 27 in Fig. 1e).

Unlike X-ray diffraction, NMR is highly sensitive to hydrogen nuclei, making it the method of choice for validating the tautomeric form of **1**. Indeed, any of the two nitrogen atoms of the pyrazole ring (labelled 5 and 6 in Fig. 1e) can be protonated in the crystalline sample. After computing ^1^H, ^13^C and ^15^N shifts for the two possible tautomers displayed in Fig. 2d and comparing them with the experimental shifts (Fig. 2e), the resulting chemical shift RMSE was found to be consistently lower for tautomer A, by a factor of 1.3 for ^1^H, 2.6 for ^13^C and 8.1 for ^15^N. This unambiguously identifies tautomer A as the crystal structure. The position of the N-H proton on the pyrazole ring is crucial in setting up amorphous structures able to describe the properties of the amorphous phase of **1**.

The atomic displacement parameter (ADP) tensors of all atoms in the structure determined by NMR crystallography were obtained as described in ref. ^32^. Simulation details are given in Supplementary Methods. Figure 2f shows the ORTEP plot of the ADP tensors corresponding to a ^1^H chemical shift RMSE of 0.34 ppm. This value corresponds to the estimated error of ^1^H chemical shifts computed with the fragment- and cluster-based approach^24^. The average value of the ADPs is 0.00025 Å^2^.

### Hydrogen bonding motifs in the amorphous phase

Knowledge of the structure of AZD5718 in the amorphous phase is key to understanding its physicochemical properties. Investigation of the amorphous structure of **1** was performed using NMR experiments combined with ShiftML predicted chemical shifts for MD ensembles.

Comparison of the proton, carbon and nitrogen NMR spectra in crystalline and amorphous **1** shown in Fig. 1a–c displays the overall broadening of the NMR signal typical of amorphous compounds. Apart from this observation, the chemical shifts do not display a significant change between the two phases of the compound. This suggests that **1** does not undergo large amplitude structural rearrangements upon transition from the crystalline to the amorphous state. The main difference between the NMR spectra of the two phases of **1** lies in the displacement of the ^1^H resonance corresponding to the proton attached to the nitrogen labelled 6 (see Fig. 1e) from 10.6 ppm in the crystalline sample to 11.8 ppm in the amorphous form. This suggests a change in the hydrogen bonding network in the structure^33^.

**Fig. 3:**
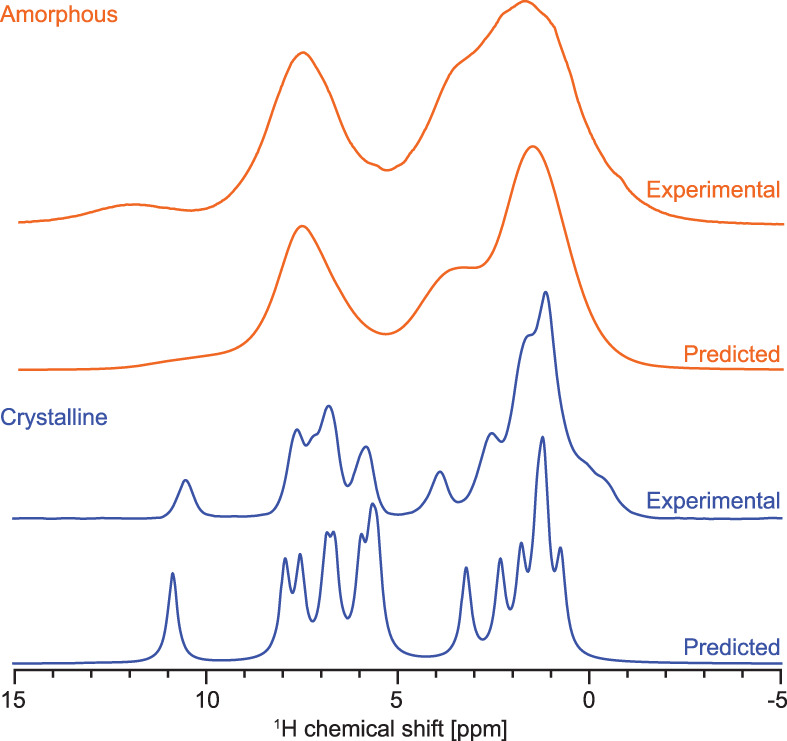
Predicted spectra. Predicted and experimental ^1^H NMR spectra of crystalline (blue) and amorphous (orange) **1**. The predicted spectrum of amorphous **1** was obtained by considering only the 4% w/w water MD simulations.

To better understand the structural differences between the amorphous and crystalline phases of **1**, we generated MD models of the amorphous structure at different hydration levels ranging from 0% to 4% (w/w) water content. This range of water content is representative of the experimental water content under real conditions, as confirmed by dynamic vapour sorption. Considering the large size of the simulation cells (128 molecules of **1** and up to 132 water molecules) and the large number of structures generated by MD, DFT computation of chemical shifts in these model systems would not be feasible. The machine learning model ShiftML was thus used to predict chemical shifts in these structures. The predicted spectra obtained for the crystalline structure and obtained by summing the spectra from 202 full cell snapshots of the 4% water MD simulations (i.e., 25,856 molecules of **1** and 26,664 water molecules) are displayed in Fig. 3. The ^1^H chemical shift RMSE obtained by comparing shifts predicted by ShiftML from the crystal structure with the experiment was found to be 0.61 ppm.

Although no clear peak is observed at 11.8 ppm for the amorphous structure, the population of predicted shifts above 11 ppm was found to increase slightly with increasing water content (see Supplementary Fig. 3). This behaviour suggests that interaction of **1** with water molecules does promote deshielding of the proton attached to the nitrogen labelled 6.

**Fig. 4:**
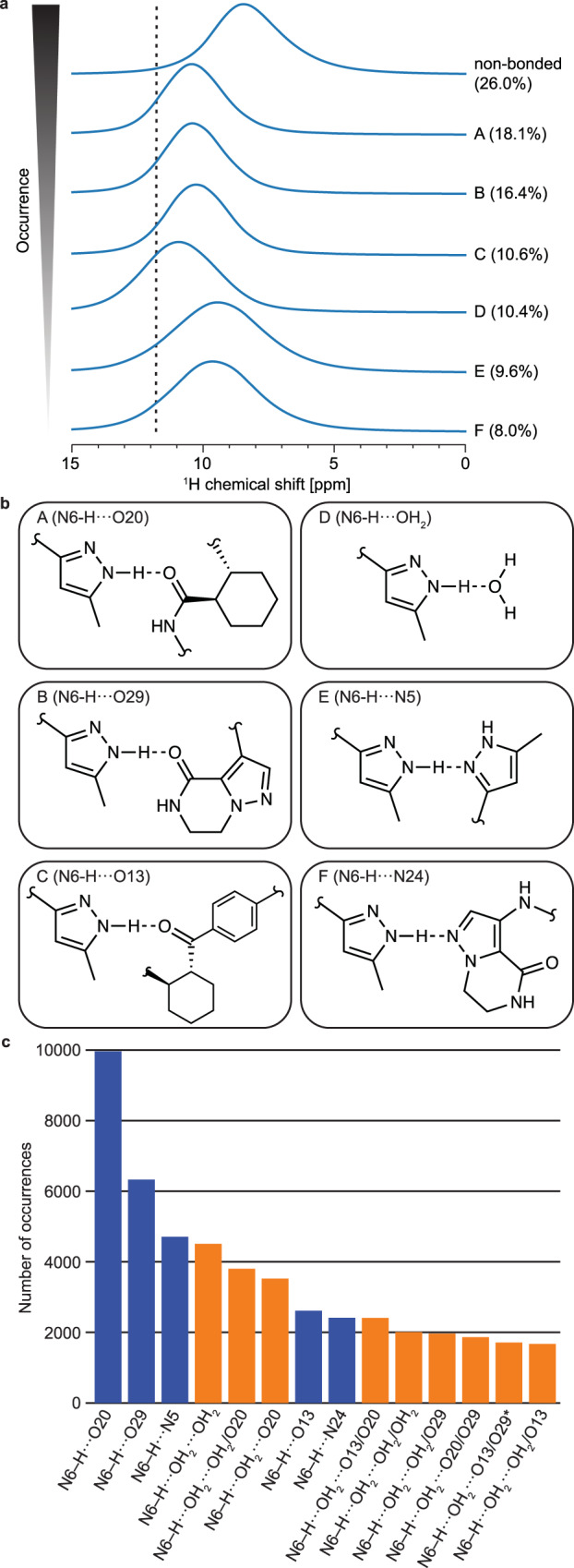
H-bonding motifs in amorphous AZD5718. **a** Predicted spectra obtained using the predicted ^1^H chemical shifts of the most often occurring N-H bonding motifs involving N6 for 11 evenly spaced snapshots of all amorphous simulations of each water content. The percentages next to the spectra denote the fraction of bonding motifs their corresponding pattern represents, including the instances where no H-bonded neighbour was identified. The dashed vertical line indicates the experimental shift observed in amorphous **1** and assigned to the proton bound to N6. **b** Hydrogen bonding motifs associated with the spectra in (**a**). **c** Number of occurrences of extended H-bonding motifs yielding a predicted chemical shift above 11 ppm for every snapshot of the 4% water simulations. Only the patterns corresponding to the top 75% of all shifts above 11 ppm were selected. The orange bars represent the bonding motifs involving water, and the blue ones correspond to the motifs that do not involve water. Two secondary neighbours from the same molecule are indicated by an asterisk. In (**b**) and (**c**), O*n* indicates the oxygen atom bonded to carbon *n*.

The predicted chemical shifts obtained were related to structural motifs in the model amorphous structures by identifying the different hydrogen bonding patterns (where the criteria are given in the methods section below) present in the structures and associated predicted ^1^H shifts of the hydrogen bond donor groups. Figure 4a displays the chemical shift distributions of the most often occurring hydrogen bonding motifs. The atom most commonly bound to the N6-H group was found to be O20 (where O20 is the oxygen bound to C20), which corresponds to the hydrogen bond found in crystalline AZD5718. This is an indication that the structure of the amorphous compound is broadly similar to that of its crystalline counterpart. Over all analysed simulation snapshots (corresponding to an average water content of 1.16% (w/w), or about 3.4 times more molecules of **1** than water), water was found to be the fourth most occurring hydrogen bonding partner to the N6-H group, and was found to lead to the most pronounced deshielding of the hydrogen bond donor proton (motif D in Fig. 4b).

**Fig. 5:**
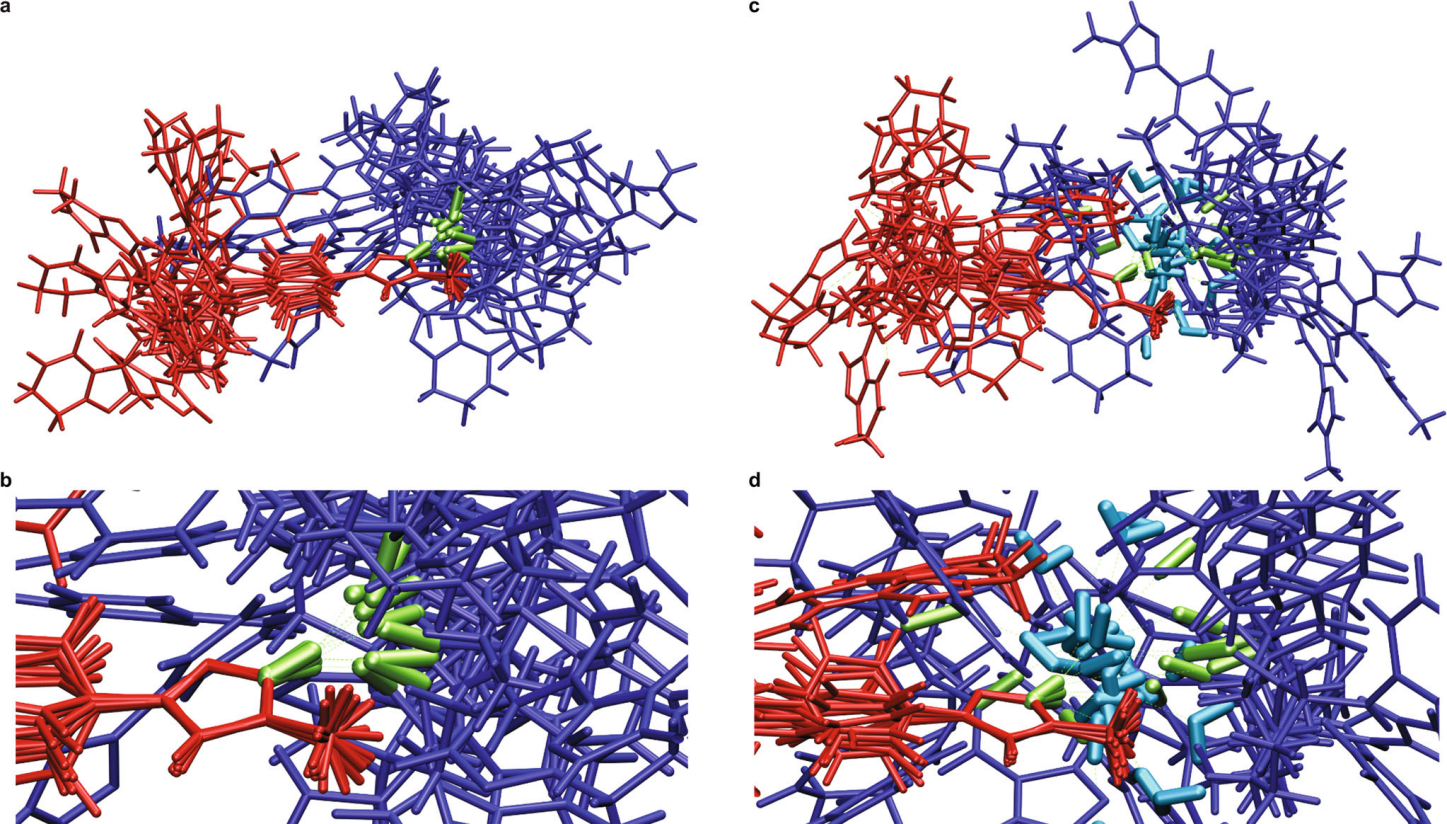
Complete structures and H-bonding motifs. **a** Superposition of 10 instances of the N6-H···O20 bonding motif. **b** close-up view of the hydrogen bonding region in (**a**). **c** Superposition of 10 instances of the N6-H···OH_2_···OH_2_/O20 bonding motif. **d** close-up view of the hydrogen bonding region in (**c**). The red molecule represents **1** bearing the hydrogen bond donor (N6-H), the dark blue molecule represents **1** bearing the hydrogen bond acceptor, water molecules are coloured in cyan and the atoms of **1** involved in the hydrogen bonding motif are coloured in green.

Because a single water molecule can form two hydrogen bonds involving its hydrogen atoms and two additional ones involving its oxygen atom, more extended hydrogen bonding motifs are likely to be observed for AZD5718 molecules bound to water. In order to investigate these extended patterns, we extracted N6-H···OH_2_ motifs yielding a chemical shift above 11 ppm in all snapshots of the amorphous 4% water MD simulations, and obtained the secondary neighbours, bonded to the water protons. We restricted this analysis to the simulations with the highest water content, as bonding of water was found to lead to the largest deshielding of the proton attached to N6 (see Fig. 4a), and as a larger number of water molecules in the simulation promotes extended hydrogen bonding motifs. Figure 4c shows the occurrences of extended hydrogen bonding patterns involving water, as well as the motifs made of pairs of H-bonded molecules of **1**, yielding a predicted shift above 11 ppm. The most often occurring pattern is the hydrogen bond present in the crystalline phase of the compound (N6-H···O20). When the H-bonded molecule is water, secondary neighbours are often found to be other water molecules, suggesting the formation of small clusters of water between AZD5718 molecules. Superpositions of ten instances of two hydrogen bonding motifs, N6-H···O20 and N6-H···OH_2_···OH_2_/O20, are shown in Fig. 5. It was observed that molecules of **1** being secondary H-bonded neighbours of N6-H generally lie away from the molecule bearing the hydrogen bond donor, indicating that steric clashes may constrain the possible geometries of hydrogen bonding in the amorphous form.

### Formation energies of intermolecular complexes

Obtaining the formation energies of the supramolecular complexes of molecules of **1** and their surroundings can help determine which hydrogen bonding pairs lead to overall more favourable intra- and intermolecular interactions. After computing the formation energies, including the conformational energy of the probe molecule, using the semiempirical DFTB3-D3H5 method, the results were gathered as a function of the hydrogen bonding motifs in which the probe molecule was involved as the hydrogen bond donor. The relative formation energy was defined as the difference between the formation energy of each instance and the mean formation energy of all instances where no hydrogen bond acceptor was found for the selected hydrogen bond donor.

**Fig. 6:**
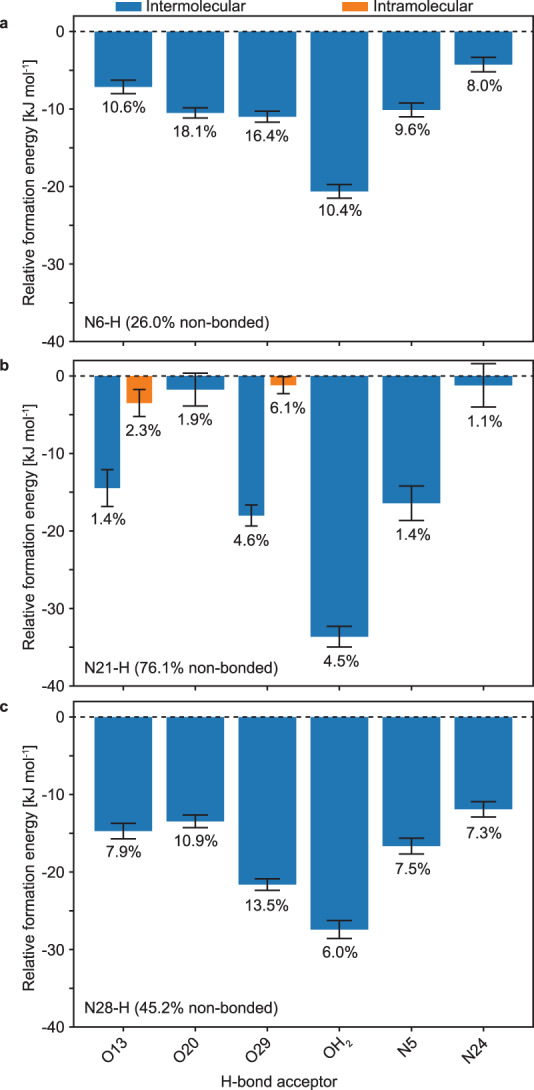
Relative formation energy of the hydrogen bonding motifs. Mean computed formation energies of intermolecular complexes for the H-bond acceptor connected to **a** N6-H, **b** N21-H or **c** N28-H of the probe molecule. The percentage under each bar indicates the fraction of the N-H group bonded to the corresponding H-bond acceptor. Only the H-bond acceptors making up at least 1% of all instances analysed are displayed. The error bars indicate the standard error of the mean of the relative formation energies.

Figure 6 shows the relative formation energy for each hydrogen bond donor and acceptor identified. Bonding of any N-H group to water was found to yield the most favourable interactions. Over all the simulation snapshots analysed for hydrogen bonding motifs, 6.1% of N21-H chemical groups were found to form an intramolecular hydrogen bond with the carbonyl labelled 29. This number may however be underestimated, as the same intramolecular hydrogen bond is found in the crystal structure, with a bond angle of 128.8°. This hydrogen bond would thus not be identified using the cutoff values selected here.

## Discussion

The atomic-level structure and hydrogen bonding patterns of the hydrated amorphous phase of AZD5718 were determined through solid-state NMR chemical shifts, MD simulations with various water contents, and machine learned chemical shifts. The chemical shifts associated with possible hydrogen bonding motifs generated from MD simulations were compared to experimental NMR spectra in order to identify the most commonly occurring intermolecular interactions in the amorphous material. Bonding of N6-H to water was found to yield the largest deshielding of the proton involved in the hydrogen bond, and best described the experimental shift observed in the amorphous sample. This intermolecular bond to water was also associated with more favourable intermolecular complex formation energies as compared to direct H-bonding between two AZD5718 molecules. These favourable water-AZD5718 interactions highlight the potential ability of water to prevent physical aging of the amorphous drug.

The combination of the three techniques presented here was crucial in elucidating the structure of this amorphous material through a large scale direct comparison of experimental chemical shifts with predicted shifts from MD structures. While solid-state NMR has already been used in tandem with MD simulations of amorphous materials, previous work have generally used molecular dynamics either to relate relative NMR peak areas to statistical ratios of different types of interactions^28,34^, or to generate conformational ensembles from which small supramolecular clusters are extracted for DFT shift computation^35^. Overall, the method presented here can be applied to a wide range of disordered organic systems to determine their complete atomic-level structures from their NMR spectra.

The structure of the crystalline form was also determined using NMR crystallography to within a positional error of 0.1 Å and was confirmed to be almost identical to the structure obtained with single crystal X-Ray diffraction.

## Methods

### NMR experiments

Both crystalline and amorphous forms of **1** were provided by AstraZeneca. The samples were stored at equilibrium with the environment at ~22 °C and 20% relative humidity prior to NMR analysis. The room temperature NMR experiments were performed on Bruker Ascend 500 wide-bore Avance III, Bruker 800 Ultrashield plus narrow-bore and 900 US^2^ wide-bore Avance Neo NMR spectrometers. DNP solid-state NMR spectroscopy experiments were performed on a 400 MHz Avance III HD Bruker spectrometer. The spectrometer is equipped with a low temperature magic angle spinning (LTMAS) 3.2 mm probe and connected through a corrugated waveguide to a 263 GHz gyrotron capable of outputting ca. 5–10 W of continuous wave microwaves. All chemical shifts were referenced to alanine. For more details including experimental setup and the sample preparation see Supplementary Methods.

### NMR crystallography

The candidate crystal structures were generated using a Monte-Carlo parallel tempering method^36^ followed by lattice energy minimisation using an internally developed force-field. The 190 most stable candidates were selected for full DFT-D optimization at the PBE level of theory. Chemical shifts for the ten lowest energy candidates were computed at the PBE0 level of theory using the fragment- and cluster-based approach developed by Hartman et al.^22–24^. The conversion from isotropic shielding to chemical shift was performed by linear regression between the obtained shieldings and experimental isotropic chemical shifts. The analysis of the positional uncertainty of the crystal structure was performed as described by Hofstetter et al.^32^ by computing shifts of perturbed crystal structures obtained through low-temperature MD simulations of candidate #1 and relating chemical shift deviations to positional deviations. Chemical shift computations were also performed on an extended set of the 81 following lowest energy candidates, but did not lead to lower shift RMSEs than candidate #1. Further computational details are given in the Supplementary Methods.

### Molecular dynamics simulation of amorphous structures

The amorphous structure of AZD5718 was modelled by carrying out MD simulations on periodic amorphous cells containing 128 molecules of **1** and a variable number of water molecules. Five cells of each water content; 0, 0.5, 1.0 and 2.0% (w/w, 0, 16, 32 and 65 water molecules in each cell, respectively), and two cells of 4% water (w/w, 132 water molecules in each cell) were generated. After equilibration for 1 ns using the canonical NVT ensemble at 298 K followed by 10 ns using the isothermal-isobaric ensemble (NPT) at 298 K and 1 bar, production simulations were carried out for 600 ns using the NPT ensemble at 298 K and 1 bar. Models of the amorphous structure were obtained by extracting 1001 evenly spaced snapshots from the last 100 ns of each MD simulation, corresponding to 100 ps time steps between the extracted snapshots. Further computational details are given in the Supplementary Methods.

### Chemical shift predictions and hydrogen bonding motifs

The predicted chemical shieldings of all snapshots extracted from the MD simulations (168,799,631 total shifts) were obtained using ShiftML version 1.2 (publicly available at https://shiftml.epfl.ch)^29,31^. The conversion from predicted shieldings to isotropic shifts is described in the Supplementary Methods.

H-bonded N-H groups were identified in 11 snapshots from each MD simulation, spaced by 10 ns each. The corresponding bonding motifs were extracted by defining hydrogen bonds as N-H···X (X = O, N) patterns with an N-H-X angle above 130° and H-X bond length shorter than 2.5 Å, typically corresponding to moderate to strong hydrogen bonds in organic solids^37^. If the first H-bonded neighbour was found to be a water molecule, then secondary water-bound neighbours were searched for using the same criteria to define hydrogen bonds.

In addition, the N-H groups yielding predicted ^1^H chemical shifts above 11 ppm were identified within each snapshot of the 4% water MD simulations (2,002 total snapshots), and the corresponding hydrogen bonding patterns were extracted as described above.

### Formation energies in the amorphous simulations

The formation energy of the intermolecular complex made of one molecule of AZD5718 and its local environment was computed for each molecule in the same 11 snapshots per simulation as used for identification of all the hydrogen bonding motifs. The environment of a molecule was defined as all molecules having at least one atom within 5 Å from any atom in the probe molecule. The formation energy was computed as the difference between the energy of the total intermolecular complex and the energy of the isolated environment. The obtained formation energy thus contains both the ground-state energy of the isolated probe molecule, which includes its conformational energy, and the interaction energy between the probe molecule and its environment. The single-point energy computations were performed at the DFTB3-D3H5 level of theory using the 3ob-3-1 parameter set and the DFTB + software version 20.1^38–43^.

## Supplementary information

Supplementary Information

## Data Availability

All data used in this study is freely available in the Materials Cloud repository, 10.24435/materialscloud:gg-mx. Source data are provided with this paper.

## Source data

Source Data
