# Supplementary material for: Structure determination of an amorphous drug through large-scale NMR predictions
Source: Nat Commun. 2021 May 20;12:2964. doi: 10.1038/s41467-021-23208-7 (PMC8137699; doi:10.1038/s41467-021-23208-7)
Supplement: Supplementary file 1 — Supplementary Information [file 41467_2021_23208_MOESM1_ESM.pdf]

Supplementary Information

Structure Determination of an Amorphous Drug through Large-Scale NMR Predictions

Cordova *et al*.

SUPPLEMENTARY METHODS

Sample preparation for DNP NMR

In DNP MAS experiments, the high thermal polarization is transferred from unpaired electrons to nuclei (typically ^{1}H) which results in enhanced NMR signals. For organic powders, this is achieved by impregnating the powdered solid with an otherwise inert polarizing solution.[1,2] **1** dissolves both in water and in most organic solvents, so most typical polarizing solutions, such as 16 mM TEKPOL in 1,1,2,2-tetrachloroethane (TCE), were found to be incompatible. *Ortho*-terphenyl was found to be a suitable non-solvent for AZD5718, and 16 mM TEKPOL in *ortho*-terphenyl 99.5%-d_{14} (OTP-d_{14}) was used as a polarization source. The sample was prepared according to the procedure described in references [3] and [4] by mixing a solid solution of 16 mM TEKPOL in OTP-d_{14} with powdered **1**, then transferring it to a sapphire rotor sealed with a PTFE insert and capped with a zirconia drive cap. The rotor was then heated at ca. 65°C in a hot water bath in order to melt the OTP and allow the liquid to impregnate the API. It was then quickly inserted into the pre-cooled LT-MAS DNP probe to rapidly freeze the sample in order for the OTP to form a glass.[4] DNP enhancements of about 5 as measured on crystalline AZD5718 signals through (^{1}H)^{13}C DNP CPMAS were obtained, which was sufficient to allow the natural abundance INADEQUATE spectra to be recorded.

NMR spectroscopy

Experiments were performed on Bruker Ascend 400 and Ascend 500 wide-bore Avance III, and on Bruker 800 Ultrashield plus narrow-bore, and 900 US^{2} wide-bore Avance Neo NMR spectrometers. The spectrometers operate at ^{1}H Larmor frequencies of 400.13, 500.43, 800.13, and 900.13 MHz respectively, and are equipped with H/X/Y 3.2 mm, H/C/N/D 1.3 mm and H/C/N 0.7 mm CPMAS probes. When the 3.2 mm probe was used, the samples were restricted to the central third of a rotor with an inner diameter of 2.2 mm, in order to maximize rf homogeneity.

DNP solid-state NMR spectroscopy experiments were performed on a 400 MHz Avance III HD Bruker spectrometer. The spectrometer is equipped with a low temperature magic angle spinning (LTMAS) 3.2 mm probe and connected through a corrugated waveguide to a 263 GHz gyrotron capable of outputting ca. 5-10 W of continuous wave microwaves.[5] The sweep coil of the main magnetic field was optimized so that the microwave irradiation gave the maximum positive proton DNP enhancement with binitroxide cross effect-based polarizing agents (e.g. AMUPOL, TEKPOL). DNP enhancements were determined based on the ratio of the area of the spectra acquired with and without microwave irradiation.

1D ^{1}H MAS NMR spectra were recorded at a temperature of 298 K using rotor spinning rates (ν_r) up to 111 kHz. 1D ^{13}C cross-polarization[6] (CP) MAS NMR spectra were acquired at 298 K with ν_r of 22 kHz. The CP contact time was 2 ms and during the signal acquisition SPINAL-64 decoupling[7] was applied with a ^{1}H rf field amplitude of 100 kHz. 1D ^{15}N CP NMR spectra were acquired at 100 K under DNP MAS conditions with $\nu_r = 12.5$ kHz for crystalline AZD5718, and similar measurements were made on amorphous AZD5718 using LT-MAS conditions (without DNP) in the same instrument. Variable amplitude cross-polarization[8] was used to transfer polarization from ^{1}H (60% to 100% ramp) to ^{15}N (constant amplitude). For the ^{15}N CPMAS spectra of crystalline AZD5718, 360 scans were acquired with DNP spaced by a recycling delay of 20 s leading to a total acquisition time of 2 h. For amorphous AZD5718, 14,720 scans were acquired without DNP, spaced by a recycling delay of 5 s leading to a total acquisition time of 21 h.

2D ^{1}H-^{13}C HETCOR experiments were carried out at 298 K using $\nu_r = 22$ kHz. 96 points were acquired in the indirect dimension with the States acquisition method,[9] and with indirect sampling intervals (Δt_1) of 96 μs. For the crystalline sample the recycle delay was 32 s ($T_1 \sim 22$ s) and 64 scans were collected for each t_1 point. For the amorphous sample the recycle delay was 4 s ($T_1 \sim 3$ s) and 769 scans were collected for each t_1 point. During t_1 100 kHz eDUMBO-1_{22} was applied to decouple the ^{1}H-^{1}H dipolar coupling,[10] and during t_2 100 kHz SPINAL-64 decoupling was applied.

The 2D ^{13}C-^{13}C refocused INADEQUATE[11,12] spectrum of crystalline **1** was acquired using DNP MAS NMR.[13] For the ^{13}C-^{13}C refocused INADEQUATE experiment, the probe was configured

into $^{1}H/^{13}C$ double resonance mode. Variable amplitude cross-polarization[8] was used to transfer polarization from ^{1}H to ^{13}C. SPINAL-64[7] heteronuclear ^{1}H decoupling with RF fields of 100 kHz was applied in all cases.

The DNP enhancement allowed to record a ^{13}C-^{13}C refocused ^{13}C-^{13}C INADEQUATE spectrum at natural abundance for **1** in about 2 days of signal averaging. Moreover, using a ^{1}H spin-lock of 30 ms between the ^{1}H excitation pulse and the CP, the otherwise dominant OTP solvent signal was efficiently removed,[14] allowing to record a 2D spectrum ^{13}C-^{13}C refocused DNP INADEQUATE clean from the solvent signal. The spectrum was acquired in about 45 h with 128 points recorded in the indirect dimension with 256 scans each separated by recycling time of 5 s. The increment in the indirect dimension was 40 μs, allowing a total indirect acquisition time of 5.12 ms using the States-TPPI method.[15] The tau period for J evolution was optimized and set to 4 ms. SPINAL-64 was used for heteronuclear decoupling.

All chemical shifts were referenced via alanine. The full set of acquisition parameters is given in Supplementary Tables **1-4**.

Solid-state NMR experimental setup

Supplementary Table 1. Experimental parameters for 1D experiments on AZD5718 form A anhydrous

	^{1}H	^{13}C	^{15}N
MAS rate	111 kHz	22 kHz	12 kHz
Recycle delay (d_1)	10 s	32 s	20 s
^{1}H to X CP			
Spin lock duration	-	2 ms	10 ms
Total acquisition time	5.5 ms	30 ms	25 ms
Dwell time	2.8 μs	13.2 μs	12.3
Number of points	1964	2268	2032
Number of scans	4	128	360
Acquisition mode	DQD	qsim	qsim

Supplementary Table 2. Experimental parameters for 2D experiments on AZD5718 form A anhydrous

	^{1}H-^{13}C HETCOR	^{13}C-^{13}C INADEQUATE
MAS rate	22 kHz	12.5 kHz
Recycle delay (d_1)	32 s	5 s
^{1}H to X CP		
Spin lock duration	0.1 ms	3 ms
Acquisition in the indirect dimension (t_1)		
Total acquisition time	4.6 ms	2.6 ms
Dwell time	96 µs	20 µs
Number of points	96	256
Acquisition in the direct dimension (t_2)		
Total acquisition time	33 ms	15 ms
Dwell time	9.9 µs	5 µs
Number of points	3328	128
Number of scans per increment	64	128
Acquisition mode	States	States-TPPI
Delay t	-	5 ms

Supplementary Table 3. Experimental parameters for 1D experiments on AZD5718 amorphous

	^{1}H	^{13}C	^{15}N
MAS rate	62.5 kHz	22 kHz	8 kHz
Recycle delay (d_1)	6.5 s	4 s	5 s
^{1}H to X CP			
Spin lock duration	-	2 ms	10 ms
Total acquisition time	8.2 ms	30 ms	25 ms
Dwell time	1.0 µs	9.9 µs	12.3
Number of points	8192	3024	2032
Number of scans	4	128	30720
Acquisition mode	DQD	qsim	qsim

Supplementary Table 4. Experimental parameters for 2D experiments on AZD5718 amorphous

	^{1}H-^{13}C HETCOR
MAS rate	22 kHz
Recycle delay (d_1)	4 s
^{1}H to X CP	
Spin lock duration	0.1 ms
Acquisition in the indirect dimension (t_1)	
Total acquisition time	4.6 ms
Dwell time	96 μs
Number of points	96
Acquisition in the direct dimension (t_2)	
Total acquisition time	33 ms
Dwell time	9.9 μs
Number of points	3328
Number of scans per increment	769
Acquisition mode	States

CSP protocol

To generate a predicted polymorph landscape for **1**, the molecular conformation determined via single-crystal XRD was optimized at the B3LYP-D3/6-31G(d,p)[16-19] level of theory in an implicit water environment using the Gaussian 09 Rev. D.01 program.[20] The media surrounding the molecule was described using the Self Consistent Reaction Field (SCRF) PCM method[21] with a dielectric constant ε set to 78.35530, as implemented in the Gaussian software. Atomic charges were obtained using the charges from electrostatic potentials using a grid-based method (CHELPG).[22] This is a slightly modified procedure compared to the previously published in-house CSP method using an internally developed force-field (AZ-FF).[23] The optimized geometry was then used in a single-point energy computation using the MacroModel program,[24] where a unique force-field for **1** was constructed. Conformational analysis was then performed within the GRACE program[25,26] in order to determine what parameters were allowed to be flexible in the molecule. For **1**, all single bonds were allowed to be rotated, and the two saturated rings were allowed to adopt different ring conformations.

Candidate crystal structures were generated in the seven most stable chiral space groups ($P2_1$, $P2_12_12_1$, P1, C2, $P2_12_12$, $P4_3$, $C222_1$) employing the GRACE machinery for a flexible conformation under Z'=1 condition. The crystal structure space was searched using a Monte-Carlo (MC) parallel tempering method[27] followed by lattice energy minimization for each polymorph using the AZ-FF force field.[23] The search was continued until the convergence criterion for statistically finding all polymorphs in the search, set to 0.7, was met.[23] Typically, 3,000 structures are kept at this stage. A structure duplicate check allowed to reduce this number to 1,000 unique structures. From these, the top 190 candidates, named #1 through #190 by increasing force field energy, were selected for full DFT-D optimization using the PBE functional[28] and Neumann-Perrin dispersion correction[25] in the VASP software.[29-32] The default PAW pseudopotentials and a 520 eV plane-wave energy cutoff were used. The ten most stable polymorphs (within 6 kJ/mol) were then selected for NMR computation. An extended set of the following 81 most stable structures (within 23.3 kJ/mol) was also selected for NMR computation, but did not lead to a better match of the experimental chemical shifts than structure #1. These 81 structures were thus not included in the set of structures used for the Bayesian analysis displayed in Fig. **2c** in the main text.

Chemical shift computation of candidate crystal structures

The proton positions of the candidates selected for NMR computations were optimized using the plane-wave DFT software Quantum ESPRESSO version 6.5.[33-35] The constrained optimizations were performed at the PBE level of theory[28] using Grimme D2 dispersion correction[36] and projector augmented wave scalar relativistic pseudopotentials with GIPAW reconstruction, H.pbe-tm-new-gipaw-dc.UPF and C.pbe-tm-new-gipaw-dc.UPF,[37] and N.pbe-n-kjpaw_psl.1.0.0.UPF and O.pbe-n-kjpaw_psl.1.0.0.UPF.[38] The wavefunction and charge density energy cutoffs were set to 60 and 240 Ry, respectively, and the relaxations were carried out without k-point.

Chemical shifts were computed for the candidate crystal structures obtained through the CSP procedure at the PBE0 level of theory[39] using the cluster- and fragment-based approach introduced by Hartman et al.[40-42] (computational details are provided in Supplementary Table **5**). Direct linear regression between the chemical shieldings computed for each candidate and the experimental chemical shifts were performed in order to obtain computed chemical shifts. The computations were run using the hybrid-many-body-interaction (HMBI) code[43,44] with Gaussian 16 Revision A.03 as the DFT engine.[45] The computed chemical shieldings σ_{calc} were converted to isotropic chemical shifts δ_{calc} through the relationship

$$\delta_{\text{calc}} = \sigma_{\text{ref}} - b\sigma_{\text{calc}} \tag{1}$$

For each candidate crystal structure, the value of σ_{ref} and b were determined by linear regression between computed and experimental shifts, permuting the ambiguously assigned shifts to obtain the lowest root-mean-square error (RMSE).

Supplementary Table 5. Cutoffs and basis sets used in the cluster/fragment DFT computations.

Cutoff description	Cutoff [Å]	Basis set
Cluster cutoff	0	
Pair-wise interaction cutoff	6	
Electrostatic embedding cutoff	30	
Basis set 1	2	6-311+G(2d, p)
Basis set 2	4	6-311G**
Basis set 3	12	6-31G

Positional uncertainty of the crystal structure

Perturbed crystal structures were obtained by performing molecular dynamics simulations of the crystal structure at 1, 5, 10, 15, 20 and 25 K. 300 ps simulations were carried out with a time step of 0.5 fs and using the canonical (NVT) ensemble, and 21 snapshots were extracted from the last 150 ps of each simulation. The force-field and parameters used are the same as the ones used to model the amorphous structure (see Section 1.8), except for the electrostatic and Van der Waals interaction cutoffs, which were set to 2.8 Å to avoid self-interaction. No constraint on the bond lengths to hydrogen was set. The correlation between chemical shift RMSD $\langle\delta\rangle$ and the average positional RMSD of atom i along the lth principal axis of its ensemble of positional deviations $\langle r_{i,l}\rangle$ was obtained by maximizing the log-likelihood between the computed correlation points and the Gaussian distribution described by Equation (2) as a function of the Gaussian parameters $\mu_{i,l}$ and $\Sigma_{i,l}$.

$$G\big(\langle r_{i,l}\rangle, \langle\delta\rangle\big) = \frac{1}{\sqrt{2\pi\Sigma_{i,l}^2\langle\delta\rangle^2}} \exp\left(-\frac{\big(\langle r_{i,l}\rangle - \mu_{i,l}\langle\delta\rangle\big)^2}{2\Sigma_{i,l}^2\langle\delta\rangle^2}\right) \tag{2}$$

The corresponding principal value of the atomic displacement parameters along the lth principal axis $U_{ii,l}$ is obtained from the variance of the Gaussian distribution (Equation (3)).

$$U_{ii,l} = \Sigma_{i,l}^{2}\langle\delta\rangle^{2} \quad (3)$$

Generation of amorphous structures

To model the amorphous structure of AZD5718, we carried out MD simulations on periodic amorphous cells with a variable number of water molecules. The atomic positions of a single molecule of **1** extracted from the crystal structure determined via single-crystal XRD were first optimized at the B3LYP-D3/6-31G(d,p)[16-19] level of theory in gas phase using the Gaussian 09 revision D.01 program.[20] Optimized coordinates and CHELPG charges were extracted from the optimization and used as input to generate amorphous cells. Materials Studio[46] together with the COMPASS-II[47] force field were used to create cubic amorphous cells of 128 molecules of **1**. Five cells of each water content; 0, 0.5, 1.0 and 2.0% (w/w, 0, 16, 32 and 65 water molecules in each cell, respectively), and two cells of 4% water (w/w, 132 water molecules in each cell) were generated. Geometries were optimized during the construction. The mean initial cell volumes were 73,004, 73,372, 73,740, 74,500, and 76,042 Å^3 for the 0, 0.5, 1, 2 and 4% water simulations, respectively.

The optimized coordinates and CHELPG charges of **1** were used as input to generate OPLS_2005[48,49] force field parameters using the Schrödinger ffld_server[50]. The "ffconv.py" tool was used to convert the topology into GROMACS format.[51] Water was treated using the TIP3P model in the MD simulations.[52]

Molecular dynamics simulation of amorphous structures

The GROMACS program (version 2016.4)[53,54] was used for all MD simulations throughout the study. The systems were initially equilibrated for 1 ns using the canonical (NVT) ensemble at 298 K. The temperature was held constant using a modified Berendsen thermostat with velocity-rescaling with a coupling constant of 0.1 ps.[55] A second equilibration was carried out for 10 ns using the isothermal-isobaric ensemble (NPT) at 298 K and 1 bar where the temperature and pressure were held constant using the velocity-rescaling thermostat with a coupling constant of 0.1 ps and a Berendsen barostat with a coupling constant of 1 ps.[55,56] Production simulations were carried out for 600 ns using the NPT ensemble at 298 K and 1 bar where the temperature and pressure were held constant using the velocity-rescaling thermostat[55] with a coupling constant of 0.1 ps and the Parrinello-Rahman barostat with a coupling constant of 4 ps.[57,58] A particle mesh Ewald scheme[59,60] was used to compute the electrostatic interactions with a 10 Å cutoff for the real space. The same cutoff was used for van der Waals interactions, with long-range dispersion correction applied to both energy and pressure. Bond lengths to hydrogens were constrained using the LINCS algorithm.[61] System trajectories were collected every 10 ps. All simulations were performed using a time step of 2 fs. Models of the amorphous structure were obtained by extracting 1001 evenly spaced snapshots from the last 100 ns of each MD simulation, corresponding to 100 ps time steps between the extracted snapshots.

Chemical shift predictions and hydrogen bonding motifs in amorphous structures

The predicted shieldings σ_{pred} obtained using ShiftML were converted to chemical shifts δ_{pred} through the relationship:

$$\delta_{\mathrm{pred}} = \sigma_{\mathrm{ref}} - b\sigma_{\mathrm{pred}} \quad (1)$$

where σ_{ref} and b were determined by minimizing the spectral contrast angle[62] between the simulated spectra, obtained by summing Lorentzian functions with a 0.3 ppm linewidth centred on the predicted shifts, and the experimental spectra. For the crystalline compound, the regression parameters were found to be $b = -0.91$ and $\sigma_{\mathrm{ref}} = 27.9$ ppm. For the amorphous form, the regression was only performed on the 4% water simulations, and applied to all other water contents. The obtained parameters are $b = -0.99$ and $\sigma_{\mathrm{ref}} = 30.9$ ppm.

Raw data

The NMR raw data are available from https://doi.org/10.24435/materialscloud:gg-mx in JCAMP-DX version 6.0 standard format and original TopSpin format. Data are made available under the license CC-BY-4.0 (Creative Commons Attribution-ShareAlike 4.0 International).

The Python scripts used to analyse NMR crystallography and MD simulation data are available from the same link and made available under the license CC-BY-4.0 (Creative Commons Attribution-ShareAlike 4.0 International).

SUPPLEMENTARY DISCUSSION

Chemical shift assignment

The ^{1}H, ^{13}C and ^{15}N resonances of AZD5718 (Fig. 1e) were assigned using one-dimensional proton, carbon and nitrogen MAS NMR experiments (Fig. 1a-c), as well as two-dimensional refocused ^{13}C-^{13}C INADEQUATE and ^{1}H-^{13}C HETCOR experiments (Fig. 1d-e). The INADEQUATE spectrum (recorded only for the crystalline form) provides the covalent connectivities between carbon atoms, indicated by red lines in Fig. 1d. The HETCOR spectrum (Fig. 1e) correlates chemical shifts of bonded carbon and hydrogen nuclei.

Chemical shift assignments of ^{1}H, ^{13}C and ^{15}N nuclei are given in Supplementary Table 6. The two protons attached to each carbon in aliphatic rings (labelled 15-18, 26 and 27 in Fig. 1e) are not equivalent, thus two values of ^{1}H chemical shifts are reported for those nuclei.

Supplementary Table 6. **Chemical shift assignment of AZD5718.** The values for inequivalent protons attached to the same carbon are indicated by a comma, and ambiguous assignments are denoted by a slash. Ambiguous assignments of carbons that were resolved using the computed shifts of structure #1 are indicated by a star.

Label	^{1}H chemical shift [ppm]	^{13}C chemical shift [ppm]	^{15}N chemical shift [ppm]
1	1.2	11.1	-
2	-	141.5	-
3	5.8	102.3	-
4	-	149.8	-
5	-	-	295.4
6	10.6	-	205.6
7	-	139.5	-
8	6.9/7.3	123.9/125.3	-
9	6.7 / 7 / 7.6	130.1/130.8	-
10	-	133.3	-
11	6.7 / 7 / 7.6	130.1/130.8	-
12	6.9/7.3	123.9/125.3	-
13	-	201.1	-
14	3.9	46.3	-
15	0.0, 1.7	31.2	-
16	-0.5, 0.8	26.6	-
17	-0.5, 0.8	26.0	-
18	1.6, 1.6	29.2	-
19	1.6	49.8	-
20	-	174	-
21	7.7	-	118.6
22	-	125.8	-
23	6.7 / 7 / 7.6	130.8	-
24	-	-	307.2
25	-	-	194.9
26	1.7, 2.7	43.5*	-
27	1.9, 2.7	40.1*	-
28	6.9	-	105.4
29	-	161.8	-
30	-	119.7	-

Comparison of the structures determined via X-ray diffraction and NMR crystallography

The crystal structure determined using single-crystal X-ray diffraction (Supplementary Fig. **1**) was compared to the structure obtained through NMR crystallography. The superposition of the two structures is shown in Supplementary Fig. **2**. The two structures were found to be highly similar except for the conformation of the bicyclo ring (on the left of Supplementary Fig. **2**).

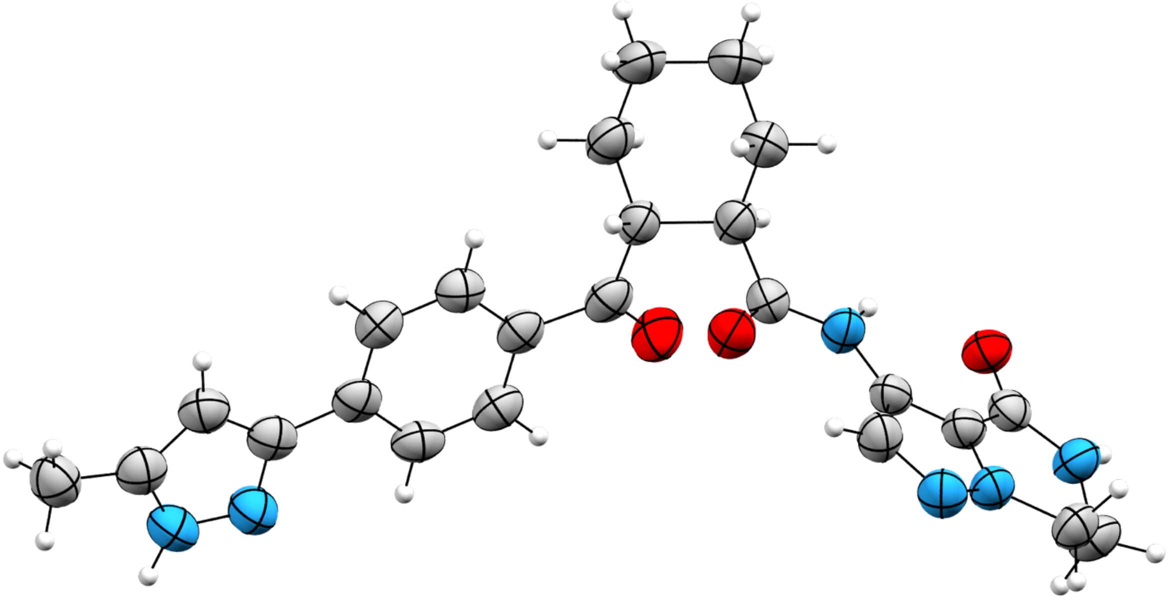

Supplementary Figure 1. **Positional uncertainty of the X-ray determined structure of AZD5718.** ORTEP plot of the heavy atom ADP tensors for the crystal structure of **1** determined using single crystal X-ray diffraction, drawn at the 90% probability level.

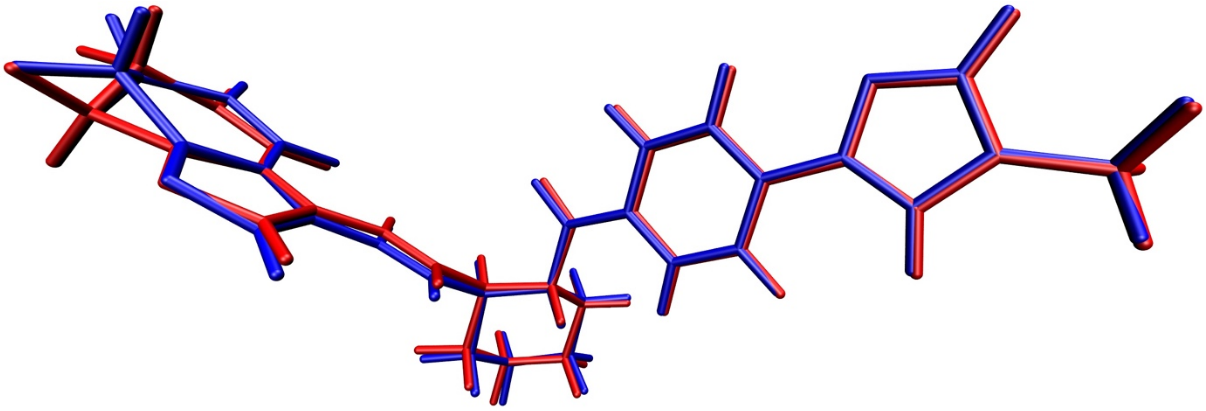

Supplementary Figure 2. **Similarity between the XRD and NMR crystallography structures.** Comparison between the structure of **1** determined using X-ray diffraction (red) and NMR crystallography (blue).

Simulated spectra of AZD5718 amorphous MD simulations with different water contents

The simulated spectrum for each water content was computed by summing Lorentzian functions centred at the predicted shifts, and with a width of 0.3 ppm. The parameters for the conversion from shielding to shift were extracted by comparing the 4.0% water simulated spectrum as described in section 1.8, and were applied to all simulations of different water contents. The spectra were normalised such that their maximum is one. Although the experimental peak observed at 11.8 ppm was not observed in the simulated spectra, a larger population of the shifts above 11 ppm was observed with increasing water content (Supplementary Fig. 3).

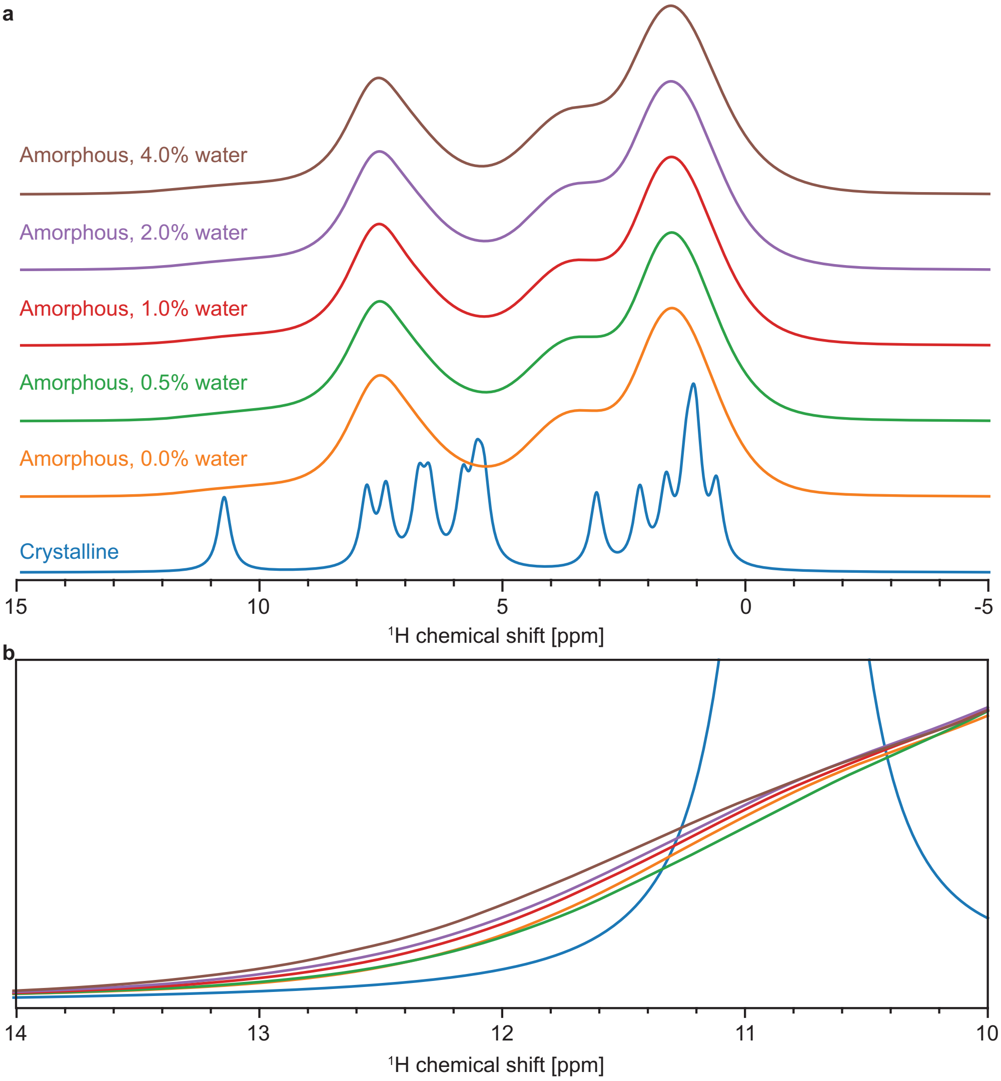

Supplementary Figure 3. **Effect of the water content on simulated ^{1}H NMR spectrum. a** Simulated ^{1}H NMR spectra of crystalline and amorphous AZD5718. **b** Close-up view of the spectra in the region between 10 and 14 ppm.

SUPPLEMENTARY REFERENCES

1 Lesage, A. *et al*. Surface Enhanced NMR Spectroscopy by Dynamic Nuclear Polarization. *Journal of the American Chemical Society* **132**, 15459-15461 (2010).

2 Zagdoun, A. *et al*. Non-aqueous solvents for DNP surface enhanced NMR spectroscopy. *Chem. Commun.* **48**, 654-656 (2012).

3 Lelli, M. *et al*. Solid-State Dynamic Nuclear Polarization at 9.4 and 18.8 T from 100 K to Room Temperature. *Journal of the American Chemical Society* **137**, 14558-14561 (2015).

4 Ong, T. C. *et al*. Solvent-Free Dynamic Nuclear Polarization of Amorphous and Crystalline ortho-Terphenyl. *J. Phys. Chem. B* **117**, 3040-3046 (2013).

5 Rosay, M. *et al*. Solid-state dynamic nuclear polarization at 263 GHz: spectrometer design and experimental results. *Phys. Chem. Chem. Phys.* **12**, 5850-5860 (2010).

6 Pines, A., Gibby, M. G. & Waugh, J. S. Proton-Enhanced Nmr of Dilute Spins in Solids. *J. Chem. Phys.* **59**, 569-590 (1973).

7 Fung, B. M., Khitrin, A. K. & Ermolaev, K. An improved broadband decoupling sequence for liquid crystals and solids. *J. Magn. Reson.* **142**, 97-101 (2000).

8 Peersen, O. B., Wu, X. L., Kustanovich, I. & Smith, S. O. Variable-Amplitude Cross-Polarization Mas Nmr. *J. Magn. Reson., Ser A* **104**, 334-339 (1993).

9 States, D. J., Haberkorn, R. A. & Ruben, D. J. A Two-Dimensional Nuclear Overhauser Experiment with Pure Absorption Phase in 4 Quadrants. *J. Magn. Reson.* **48**, 286-292 (1982).

10 Elena, B., de Paepe, G. & Emsley, L. Direct spectral optimisation of proton-proton homonuclear dipolar decoupling in solid-state NMR. *Chem. Phys. Lett.* **398**, 532-538 (2004).

11 Bax, A., Freeman, R. & Frenkiel, T. A. An Nmr Technique for Tracing out the Carbon Skeleton of an Organic-Molecule. *Journal of the American Chemical Society* **103**, 2102-2104 (1981).

12 Lesage, A., Auger, C., Caldarelli, S. & Emsley, L. Determination of through-bond carbon-carbon connectivities in solid-state NMR using the INADEQUATE experiment. *Journal of the American Chemical Society* **119**, 7867-7868 (1997).

13 Rossini, A. J. *et al*. Dynamic Nuclear Polarization Surface Enhanced NMR Spectroscopy. *Acc. Chem. Res.* **46**, 1942-1951 (2013).

14 Yarava, J. R., Chaudhari, S. R., Rossini, A. J., Lesage, A. & Emsley, L. Solvent suppression in DNP enhanced solid state NMR. *J. Magn. Reson.* **277**, 149-153 (2017).

15 Marion, D., Ikura, M., Tschudin, R. & Bax, A. Rapid recording of 2D NMR spectra without phase cycling. Application to the study of hydrogen exchange in proteins. *J. Magn. Reson.* **85**, 393-399 (1989).

16 Becke, A. D. Density-functional thermochemistry. III. The role of exact exchange. *J. Chem. Phys.* **98**, 5648-5652 (1993).

17 Stephens, P. J., Devlin, F. J., Chabalowski, C. F. & Frisch, M. J. Ab-Initio Calculation of Vibrational Absorption and Circular-Dichroism Spectra Using Density-Functional Force-Fields. *J. Phys. Chem.* **98**, 11623-11627 (1994).

18 Grimme, S., Antony, J., Ehrlich, S. & Krieg, H. A consistent and accurate ab initio parametrization of density functional dispersion correction (DFT-D) for the 94 elements H-Pu. *J. Chem. Phys.* **132**, 154104 (2010).

19 Rassolov, V. A., Ratner, M. A., Pople, J. A., Redfern, P. C. & Curtiss, L. A. 6-31G*basis set for third-row atoms. *J. Comput. Chem.* **22**, 976-984 (2001).

20 Gaussian 09 v. Revision D.01 (Gaussian, Inc., Wallingford CT, 2016).

21 Tomasi, J., Mennucci, B. & Cammi, R. Quantum mechanical continuum solvation models. *Chem. Rev.* **105**, 2999-3093 (2005).

22 Breneman, C. M. & Wiberg, K. B. Determining Atom-Centered Monopoles from Molecular Electrostatic Potentials - the Need for High Sampling Density in Formamide Conformational-Analysis. *J. Comput. Chem.* **11**, 361-373 (1990).

23 Broo, A. & Nilsson Lill, S. O. Transferable force field for crystal structure predictions, investigation of performance and exploration of different rescoring strategies using DFT-D methods. *Acta Crystallogr. B* **72**, 460-476 (2016).

24 MacroModel v. Release 2017-3 (Schrodinger, LLC, New York, NY, 2016).
25 Neumann, M. A. & Perrin, M. A. Energy ranking of molecular crystals using density functional theory calculations and an empirical van der Waals correction. *J. Phys. Chem. B* **109**, 15531-15541 (2005).
26 Neumann, M. A., Leusen, F. J. J. & Kendrick, J. A major advance in crystal structure prediction. *Angew. Chem. Int. Ed.* **47**, 2427-2430 (2008).
27 Hukushima, K. & Nemoto, K. Exchange Monte Carlo method and application to spin glass simulations. *J. Phys. Soc. Jpn.* **65**, 1604-1608 (1996).
28 Perdew, J. P., Burke, K. & Ernzerhof, M. Generalized Gradient Approximation Made Simple. *Phys. Rev. Lett.* **77**, 3865-3868 (1996).
29 Kresse, G. & Hafner, J. Ab initiomolecular dynamics for liquid metals. *Phys. Rev. B* **47**, 558-561 (1993).
30 Kresse, G. & Hafner, J. Ab initiomolecular-dynamics simulation of the liquid-metal–amorphous-semiconductor transition in germanium. *Phys. Rev. B* **49**, 14251-14269 (1994).
31 Kresse, G. & Furthmuller, J. Efficiency of ab-initio total energy calculations for metals and semiconductors using a plane-wave basis set. *Comput. Mater. Sci.* **6**, 15-50 (1996).
32 Kresse, G. & Furthmuller, J. Efficient iterative schemes for ab initio total-energy calculations using a plane-wave basis set. *Phys. Rev. B* **54**, 11169-11186 (1996).
33 Giannozzi, P. *et al.* QUANTUM ESPRESSO: a modular and open-source software project for quantum simulations of materials. *J. Phys.: Condens. Matter* **21**, 395502 (2009).
34 Giannozzi, P. *et al.* Advanced capabilities for materials modelling with QUANTUM ESPRESSO. *J. Phys.: Condens. Matter* **29**, 465901 (2017).
35 Giannozzi, P. *et al.* Quantum ESPRESSO toward the exascale. *J. Chem. Phys.* **152**, 154105 (2020).
36 Grimme, S. Semiempirical GGA-type density functional constructed with a long-range dispersion correction. *J. Comput. Chem.* **27**, 1787-1799 (2006).
37 From https://sites.google.com/site/dceresoli/pseudopotentials
38 Dal Corso, A. Pseudopotentials periodic table: From H to Pu. *Computational Materials Science* **95**, 337-350 (2014).
39 Adamo, C. & Barone, V. Toward reliable density functional methods without adjustable parameters: The PBE0 model. *J. Chem. Phys.* **110**, 6158-6170 (1999).
40 Hartman, J. D. & Beran, G. J. Fragment-Based Electronic Structure Approach for Computing Nuclear Magnetic Resonance Chemical Shifts in Molecular Crystals. *J. Chem. Theory Comput.* **10**, 4862-4872 (2014).
41 Hartman, J. D., Kudla, R. A., Day, G. M., Mueller, L. J. & Beran, G. J. Benchmark fragment-based (1)H, (13)C, (15)N and (17)O chemical shift predictions in molecular crystals. *Phys. Chem. Chem. Phys.* **18**, 21686-21709 (2016).
42 Hartman, J. D., Monaco, S., Schatschneider, B. & Beran, G. J. Fragment-based (13)C nuclear magnetic resonance chemical shift predictions in molecular crystals: An alternative to planewave methods. *J. Chem. Phys.* **143**, 102809 (2015).
43 Beran, G. J. O. Approximating quantum many-body intermolecular interactions in molecular clusters using classical polarizable force fields. *J. Chem. Phys.* **130**, 164115 (2009).
44 Beran, G. J. O. & Nanda, K. Predicting Organic Crystal Lattice Energies with Chemical Accuracy. *J. Phys. Chem. Lett.* **1**, 3480-3487 (2010).
45 Gaussian 16 v. Revision A.03 (Gaussian, Inc., Wallingfort CT, 2016).
46 BIOVIA Materials Studio v. Release 2017 (BIOVIA, Dassault Systèmes, San Diego, 2017).
47 Sun, H. *et al.* COMPASS II: extended coverage for polymer and drug-like molecule databases. *J. Mol. Model.* **22**, 47 (2016).
48 Jorgensen, W. L., Maxwell, D. S. & TiradoRives, J. Development and testing of the OPLS all-atom force field on conformational energetics and properties of organic liquids. *J. Am. Chem. Soc.* **118**, 11225-11236 (1996).
49 Banks, J. L. *et al.* Integrated modeling program, applied chemical theory (IMPACT). *J. Comput. Chem.* **26**, 1752-1780 (2005).
50 ffld_server v. Release 2017-3 (Schrodinger, LLC, New York, NY, 2017).

51 Frolov, A. I. & Kiselev, M. G. Prediction of Cosolvent Effect on Solvation Free Energies and Solubilities of Organic Compounds in Supercritical Carbon Dioxide Based on Fully Atomistic Molecular Simulations. *J. Phys. Chem. B* **118**, 11769-11780 (2014).
52 Jorgensen, W. L., Chandrasekhar, J., Madura, J. D., Impey, R. W. & Klein, M. L. Comparison of Simple Potential Functions for Simulating Liquid Water. *J. Chem. Phys.* **79**, 926-935 (1983).
53 Van der Spoel, D. *et al.* GROMACS: Fast, flexible, and free. *J. Comput. Chem.* **26**, 1701-1718 (2005).
54 Abraham, M. J. *et al.* GROMACS: High performance molecular simulations through multi-level parallelism from laptops to supercomputers. *SoftwareX* **1-2**, 19-25 (2015).
55 Bussi, G., Donadio, D. & Parrinello, M. Canonical sampling through velocity rescaling. *J. Chem. Phys.* **126**, 014101 (2007).
56 Berendsen, H. J. C., Postma, J. P. M., Vangunsteren, W. F., Dinola, A. & Haak, J. R. Molecular-Dynamics with Coupling to an External Bath. *J. Chem. Phys.* **81**, 3684-3690 (1984).
57 Parrinello, M. & Rahman, A. Polymorphic Transitions in Single-Crystals - a New Molecular-Dynamics Method. *J. Appl. Phys.* **52**, 7182-7190 (1981).
58 Nose, S. & Klein, M. L. Constant Pressure Molecular Dynamics for Molecular Systems. *Mol. Phys.* **50**, 1055-1076 (1983).
59 Darden, T., York, D. & Pedersen, L. Particle Mesh Ewald - an N.Log(N) Method for Ewald Sums in Large Systems. *J. Chem. Phys.* **98**, 10089-10092 (1993).
60 Essmann, U. *et al.* A Smooth Particle Mesh Ewald Method. *J. Chem. Phys.* **103**, 8577-8593 (1995).
61 Hess, B., Bekker, H., Berendsen, H. J. C. & Fraaije, J. G. E. M. LINCS: A linear constraint solver for molecular simulations. *J. Comput. Chem.* **18**, 1463-1472 (1997).
62 Wan, K. X., Vidavsky, I. & Gross, M. L. Comparing similar spectra: From similarity index to spectral contrast angle. *Journal of the American Society for Mass Spectrometry* **13**, 85-88 (2002).
